# *Aspergillus* Is Inhibited by *Pseudomonas aeruginosa* Volatiles

**DOI:** 10.3390/jof6030118

**Published:** 2020-07-25

**Authors:** Hasan Nazik, Gabriele Sass, Eric Déziel, David A. Stevens

**Affiliations:** 1 California Institute for Medical Research, San Jose, CA 95128, USA; hasannazik01@gmail.com (H.N.); gabriele.sass@cimr.org (G.S.); 2 Institut National de la Recherche Scientifique, Institut Armand-Frappier, Laval, QC H7V 1B7, Canada; eric.deziel@iaf.inrs.ca; 3 Division of Infectious Diseases and Geographic Medicine, Department of Medicine, Stanford University School of Medicine, Stanford, CA 94305, USA

**Keywords:** *Pseudomonas*, *Aspergillus*, volatiles

## Abstract

Background: *Pseudomonas aeruginosa* (Pa) and *Aspergillus fumigatus* (Af) compete with each other for nutrients and survival in natural environments, and have been extensively studied because of their intermicrobial interactions in the human microbiome. These are the principal microbes infecting immunocompromised patients and persons with cystic fibrosis, particularly the airways. These intermicrobial studies have largely been conducted in liquid medium or on agar, and thus focus on soluble or diffusible microbial products. Several key inhibitory molecules were defined in such studies. Methods: in the present report, we examine several methodologies which can be conveniently used to study the interaction of microbial volatiles, including capture methods and kinetics. Results: Pa volatiles inhibit Af, and the inhibitory mechanism appears to be the incorporation of the inhibitory molecules into the substrate nourishing the Af, rather than directly onto Af structures. We define by mass spectroscopy some specific volatile Pa products that can inhibit Af. Some of these molecules are selected for interest by the study of gene deletion mutants, producing a few Pa strains that were impaired in inhibition. We presumed the volatiles of these latter strains could be excluded from the search for inhibitors. Conclusion: the Pa inhibition of Af via a gaseous phase could be critical components in their competition, particularly in airways, where more direct contact may not be extensive.

## 1. Introduction

*Pseudomonas* and *Aspergillus* are ubiquitous micro-organisms inhabiting soil, water, plants, and the human microbiota [1,2]. Clinicians are concerned about their invasion of the airways of immunocompromised, particularly neutropenic, patients and persons with cystic fibrosis (CF) [3,4,5,6,7,8,9,10]. In those locales, infections with *P. aeruginosa* (Pa) and *A. fumigatus* (Af) have the most devastating consequences of any bacterium and fungus, respectively [11,12,13,14,15,16,17,18,19,20]. Both organisms have the ability to form colonies in biofilm, which protects them [21,22,23,24,25,26,27,28,29,30,31,32,33,34]. There is evidence that co-infection with those micro-organisms aggravates pulmonary pathology, which is particularly documented in CF [35]. For this reason, extensive research has proceeded to study their microbial interactions, where they may compete against each other as they do in the environment [36,37,38,39,40,41,42,43,44,45,46,47]. That research has examined their interactions in vitro and has defined microbial weaponry employed in their competition [45,48,49,50,51,52,53,54,55,56]. Such studies have largely utilized co-cultures in liquid medium, or sometimes on agar. Almost all the studies to date of the interaction of these pathogens have thus examined soluble microbial products or those diffusible in liquid or agar. However, communication via volatiles could be very important, as a microbiome could be affected by distant actors distributing their vapors, particularly in the airways. In the present studies, we examine intermicrobial interaction via volatiles, document various methodologies for such studies, and define some Pa candidate molecules that could explain Pa successes in its competition with Af.

## 2. Materials and Methods

### 2.1. Materials

All the chemicals were purchased from Sigma-Aldrich (St. Louis, MO, USA). All the commercial agars and RPMI-1640 medium were purchased from Beckton Dickinson (Sparks, MD, USA). The Bacto agar used for growth studies was obtained from Carolina Biological Supply Co., Burlington, NC. The RPMI agar was prepared; briefly, 7.5 gm of Bacto agar in 100 mL of distilled water was autoclaved and mixed with 400 mL of prewarmed RPMI-1640 medium. The agar (20 mL) was poured into petri dishes. Fe-depleted agar was prepared by formulating potato dextrose agar (PDA) in the presence of ascorbate and ferrozine, as we previously described [54]. For all the agar studies, 100 or 40 mm-diameter plastic Petri dishes were used (E and K Scientific, Santa Clara, CA, USA).

### 2.2. Isolates

In determining the activity of volatiles with Af as a target, we desired to have confirmation by demonstrations with four Aspergillus wild types: AF 293 (ATCC MYA-4609), 10AF (ATCC 90240), CBS 144-89, and *A. flavus* (CIMR 10-103). The Af strain CBS 144-89 was kindly donated by the investigators from the Institute Pasteur [57]. Clinical isolates from respiratory cultures from persons with cystic fibrosis were obtained after written informed consent for the biobanking and subsequent use of the patients’ specimens was obtained and approved by the Stanford Univ. Institutional Review Board. No patient names are associated with any of the organisms [45].

PA14 (genome published) [58], PAO1 (ATCC 15692), PAK (ATCC 25102), and the clinical isolate Pa10 [40] are the wild-type Pa strains studied. The PA14 mutants studied here, their descriptions and phenotypes, and the references for production were published previously [45] (Supplementary Table S1). The mutants of PAO1 background, *pvdD*-, *lasR*-/*rhlR*-, *pqsA*-, *coaB*, and the PAO728 mutant were kindly provided by P. R. Secor, Departments of Microbiology and Medicine, University of Washington, Seattle, WA, USA [59].

The use of all the micro-organisms in our laboratory was approved by the CIMR Biological Use Committee (approval no. 001-03Yr. 14 June 2020).

### 2.3. Methods for Pseudomonas Volatile Production

**Figure 1:**
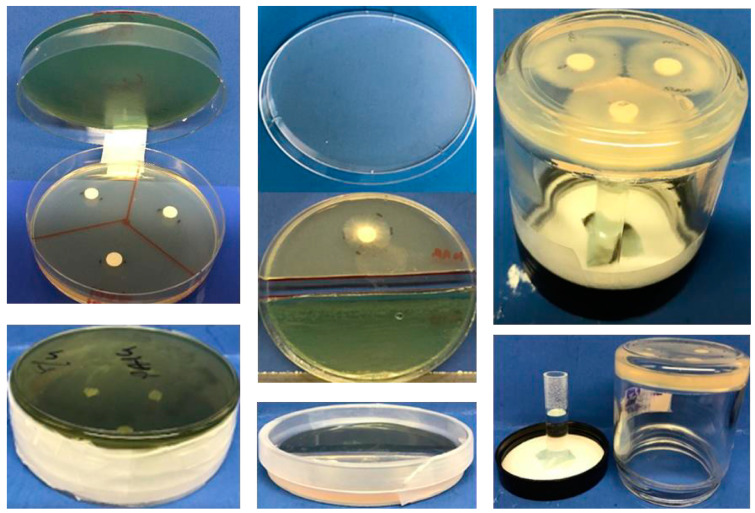
(**Left**) upper and lower, the double plate method. In this example, 3 discs can be inoculated with Af, and 3 different measurements are performed after incubation with a *Pseudomonas aeruginosa* (Pa) inoculum placed on (in this example) the upper plate. (**Middle**) upper and lower, the single plate method. Af is demonstrated growing on half the agar, while the other half can be inoculated with Pa. (**Right**) jar method. A vial containing liquid (in this example) containing a test article can be placed on the lid with an adhesive, whereas a layer of agar in the jar is inoculated with Af on the discs. Alternatively, the lid could contain agar inoculated with Pa and the lid could be screwed on loosely or tightly, depending on the conditions desired.

Double plate method: Two agar plates were used in this method. A Pa (40 µL, 10^9^/mL) suspension was inoculated in one plate and spread on the surface of the plate using a sterile plastic loop. Three sterile paper discs (Beckton, Dickinson and Company, Sparks, MD, USA) were put on the second plate, and 10 µL of Af suspension (5 × 10^4^ conidia/mL) (in all the experiments with conidia, ungerminated conidia were used) was inoculated on each disc. The lids of the plates were discarded, and the bottom parts of the plates were sealed to each other with parafilm and tape (Figure 1). The Pa was not inoculated on control plates. The plates were incubated at 48 h, 37 °C. At the end of incubation, the area of Af growth in sq. mm was calculated and compared.

This method was also used to capture Pa-derived volatiles in agar. An uninoculated agar plate was incubated with a plate inoculated with Pa, as described above, for 48 h. Following incubation, the Pa-carrying plates were removed, filter disks were placed on the exposed agar and inoculated with 10 µL of Af suspension (5 × 10^4^ conidia/mL), and the sealed plates were incubated for an additional 48 h. In a variation of this method, 4 cm agar plates were inverted, 10 mm filter disks were placed in the lids, and they were inoculated with 100 µL of volatile substances or controls. The agar plates were exposed at room temperature for 90 min. The lids were replaced, one new sterile filter disk was placed on each exposed agar, they were inoculated with 10 µL of Af suspension (5 × 10^4^ conidia/mL), and the sealed plates were incubated at 37 °C for 48 h.

Single plate method: A 3–10 mm strip of agar in the middle of an 8 cm-diameter agar plate was cut along a diameter using a sterile scalpel and removed to separate the plate into 2 semicircular noncontiguous agar halves. A Pa suspension (40 µL from 10^9^/mL) was inoculated on one part and spread on the surface of the agar using a sterile plastic loop. One sterile paper disc was placed on the second part, and 10 µL of Af suspension (5 × 10^4^ conidia/mL) was inoculated on the disc. Pa was not inoculated on the control plates (Figure 1). The plates were sealed and incubated at 37 °C for 48 h, then the *Aspergillus* colony was measured.

Vial in jar method: After autoclave sterilization, 10 mL of agar was poured into 4 oz glass jars and left at room temperature for solidifying the agar. Then, three 6 mm sterile paper discs were placed onto each agar, and 10 µL of Af suspension (5 × 10^4^ conidia/mL) was inoculated on each disc. A 5 mL vial was attached to the lid of a jar using a drop of hot silicone glue. The test compounds were placed into the glass vials. With the top of the jar lid resting on a firm surface, the jars with the agar and the lid were screwed together, kept inverted, and sealed with tape. Additionally, the lids were sealed with parafilm (Figure 1). The jars were incubated at 37 °C for 48 h.

Plate in plate method: the method used was as described previously [57].

The 96-well plate method: 75 µL of Pa suspension (2.5 × 10^7^/mL) in RPMI or TSB was added to the wells of the outer two columns and rows of 96-well plates. Af suspension (75 µL, 10^4^ conidia/mL) in RPMI was placed into the inner wells of the plates. The plates were sealed and incubated at 37 °C for 40 h. After incubation, an XTT assay was performed to measure the Af metabolic activity as described previously [40].

### 2.4. Methods for Pseudomonas Volatile Transfer

Syringe method: After incubation, the air was aspirated from the double plates by piercing the parafilm wrap with a needle. Each air sample was injected into rubber-stopper sealed glass vials, which contained an Af inoculum (10 µL from 5 × 10^4^ conidia/mL) on paper discs on TSA.

Air pump method: An air pump model was designed that transferred volatiles from double plates to glass vials. The Pa suspension (40 µL from 10^9^/mL) was inoculated on two TSA agar plates, and the plates were combined as described. As controls, TSA plate combinations with no Pa inoculation and double plate setups with no TSA or Pa were used. The double plates were sealed and incubated at 37 °C for 48 h. After incubation, the air in the double plates was transferred into vials by using a one-way air pump. The pump was set up using common laboratory materials such as clamps, tubes, electrical tape, vinyl tubing, and syringes. Silicon glue was heated and applied externally to any possible leakage sites. In this model, the volatiles in the double plate were aspirated and transferred into the glass vial directly. More specifics about the operational details are given in the figure legends and are illustrated.

Trapping volatiles: TSA, TSB, PBS, RPMI, de-ionized water, or normal saline (trapping materials) were exposed to Pa in double plates, and the trapping materials then exposed to Af plates in a second double plate step, as will be described.

### 2.5. Preparation of Samples for Mass Spectroscopy Analysis

Water samples exposed to Pa volatiles: Three double plates were used. The Pa suspension (40 µL from 10^9^/mL) was inoculated on TSA agar and combined with a petri dish with 15 mL of sterile deionized water (test article). As double plate controls, uninoculated TSA combined with 15 mL of sterile deionized water or 15 mL of sterile deionized water with an empty plate were used. The double plates were sealed and incubated at 37 °C for 72 h. After incubation, the water was aspirated and subjected to mass spectrometry.

Live cell samples: After the autoclave sterilization of TSA, 5 mL of agar was put in mass spectroscopy vials. Following solidification, the Pa suspensions (40 µL from 10^9^/mL) were inoculated onto the agars. The vials were closed with rubber stoppers and sealed with parafilm. As controls, vials were prepared with TSA without Pa inoculation and vials were prepared with no TSA and Pa. The vials were incubated at 37 °C for 48 h, and the volatiles from these vials were directly used for mass spectroscopy.

### 2.6. Identification of Volatiles

The Agilent (Santa Clara, CA, USA) model 6890/5973 was used for the headspace gas chromatography (GC)-mass spectrometry (MS), as per the manufacturer’s instructions and as detailed elsewhere [60]. In brief, the samples were heated and the volatile headspace gases were exposed to extraction via solid phase micro-extraction onto a silica fiber. The fiber was then inserted into the injection port of the gas chromatograph, where the volatiles were then thermally desorbed and analyzed by GC and MS, where the GC resolved the components based on volatility and the MS ionized the resolved components and detected them via the mass/charge ratio in the mass analyzer. The resultant peaks were referenced to a known amount of external standard semi-quantitatively.

### 2.7. Statistical Analysis

The results were analyzed with a Student’s *t*-test if the two groups were compared and a 1-way analysis of variance (ANOVA) combined with Tukey’s post-test for multiple comparisons. All the data are expressed as the mean ± the standard deviation. The data reported as percentages of the control value were compared with a Student’s *t*-test after the arcsin transformation of the proportions; these data are presented as percentages. Assays used 4–8 replicate wells for each group studied for statistical purposes, and the number of replicates in each experiment is specified in the figures. The representative experiments shown are the most complete of a series of experiments; the conclusions and statistics for the corroborating experiments are given.

## 3. Results

### 3.1. Effect of Assay Methods

We performed experiments to determine the abilities of the different methods to demonstrate the inhibitory effects of Pa volatiles on Af (10AF). In five experiments (Pa10, TSA agar), we showed that the double plate method was more sensitive for detecting inhibition than the single plate method (Figure 1). The jar method had the same sensitivity as the double plate method.

The 96-well plate method is a miniature version for testing the ability of Pa on agar to produce volatiles affecting Af on agar (not shown, layout described in Methods). It enables multiple wells of Pa and Af to be prepared, examined, and scored on the same plate. The inhibition of Af was clear (*p* < 0.001, comparison with another plate that omitted the Pa). Thus, interested investigators have four methods by which the phenomenon of Pa inhibiting Af with no direct contact between the microbes can be manipulated for further studies, which was the purpose of these initial studies.

Positing that the inhibitory volatiles could be heavier or lighter than air, affecting the results, to assure optimal testing we needed to know whether the position of the two plates in the double plate method affected the results. In two experiments (Pa10, TSA, or SDA), we found that it made no difference if the Pa plate was on the top and the Af (10 AF) plate was on the bottom, or vice versa. Thus, the inhibitory vapors diffuse freely up or down, as studied in the double plate environment.

### 3.2. Effect of Agar Substrates on Production of, and Assay of, Pa Volatiles

We tested in five experiments TSA, SDA, PDA, and RPMI agar in all possible combinations (the 16 possible combinations of these four agars) as the substrates for plating Pa (Pa10) or Af (10AF) in the double plate method. We found that the Af inhibition was negligible if the Pa was plated on RPMI agar, regardless of the four agars tested for the Af, possibly related to the apparently inferior Pa growth on RPMI agar. The Pa on PDA agar had similar results to those just described on RPMI, but to a lesser extent and less consistently. In contrast, any combinations of TSA or SDA for the Pa or Af did not affect the inhibition of Af by Pa, nor the use of RPMI agar or PDA for only the Af plate of the pair.

In two experiments, we found that the Pa volatile production measured by Af inhibition was inferior when TSB was substituted for TSA, even though the Pa growth on both was robust and quantitatively similar. Removing the iron [54] from TSA (comparing the iron-depleted TSA to TSA) also did not affect the volatile production by Pa, suggesting that iron metabolism is not critical for the Pa production of volatiles in our setting.

It was necessary to prove that the plastic plates with agar themselves emitted no Af-inhibitory volatiles [61]. In four experiments, TSA, SDA, PDA, LB, RPMI agar without Pa, a plastic or glass plate without agar, or an empty space where a second plate would have been were linked in the double plate method to the Af on TSA. There was no inhibition of Af by any putative volatiles from any agar, or from the plastic plate itself. In addition, the comparison of Af with the Pa plates in prior experiments with all the controls with no Pa now mentioned did not indicate any stimulation by Pa of Af growth, in contrast to what has been reported [57,62].

### 3.3. Inhibition Occurs with Several Pa or Af Strains

To assess whether inhibition could be related to only the micro-organisms studied in detail above, we compared (double plate method, TSA agars) the Pa clinical strain Pa10 and the Pa laboratory strains PAO1, PA14, or PAK with the Af strain 10AF or the reference strain AF293, and found no differences in any combination of Pa-Af in the inhibition we described above.

### 3.4. On the Mechanism of Inhibition

#### 3.4.1. Inhibition Is Proportional to Pa Inoculum

When 40 μL of 10-fold dilutions of Pa10 inocula 10^3^ to 10^9^ CFU/mL were plated on TSA, the inhibition of 10AF on TSA was dose-responsive from 10^3^ to 10^6^/mL, and there was no Af growth with >10^6^/mL Pa. This was confirmed in a second experiment using TSB for Pa growth.

#### 3.4.2. Studies of Time of Exposure, Reversibility of Inhibition

**Figure 2:**
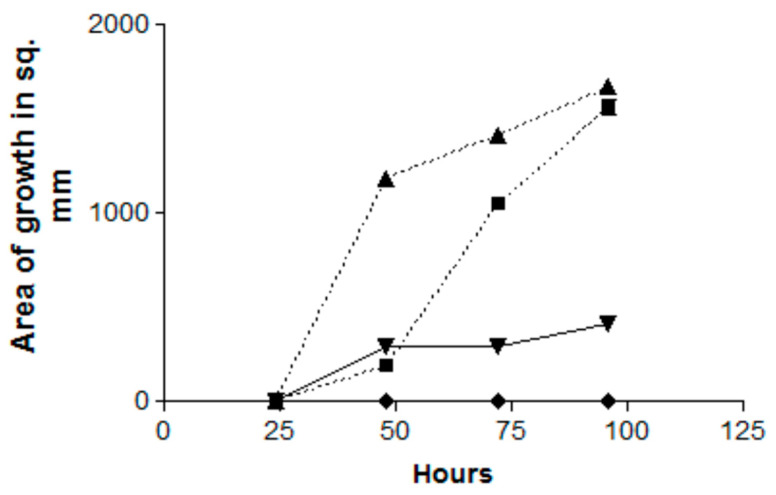
Effect of the time of exposure on the inhibition by Pa volatiles (Pa10) of Af (AF10), and the reversibility of the inhibition. Double TSA plate method. Triangles, Af control (no Pa exposure); squares, Pa plate only present for the first 24 h; inverted triangles, Pa plate presented only after 24 h; diamonds, exposure of Af to Pa for the full 96 h. The squares show escape from inhibition after 48 h. The inverted diamonds show start of release from inhibition, but the growth is then halted.

In the first of these experiments, a Pa plate was present only for the first 24 h, then was substituted by an uninoculated TSA plate, or the Pa plate was substituted for an uninoculated TSA plate at 24 h. Those two conditions were compared to a Pa plate present for a full 96 h (positive control), or only a 96 h exposure to a TSA plate that was not inoculated with Pa (negative control) (Figure 2). The positive control prevented any Af growth for the 96 h. If the Pa plate was present for only the first 24 h, Af began to grow during the next 24 h and then continued to escape inhibition, so that the Af growth attained the same amount at 96 h as the Af inoculum that had never been co-incubated with Pa (the negative control) did. If the Pa plate was introduced only after 24 h, there was some Af growth during the next 24 h but then no further Af growth (Figure 2).

In a second experiment with identical controls but incubation for 144 h, the effect of introducing the Pa plate only after 24 h had the identical total inhibition effect and this lasted until 144 h; when a Pa plate was introduced only after 48 h, there was limited Af growth for the next 24 h but then no further growth.

Thus, in summary, the inhibition by Pa volatiles is proportional to the time of exposure; the inhibition is reversible (suggesting a fungistatic effect). Moreover, the inhibition can start even after the Af has started growing (mycelial phase) in the absence of an inhibiting Pa plate.

#### 3.4.3. Continuous Production of Pa Volatiles in the Presence of Af is not Necessary for Inhibition

**Figure 3:**
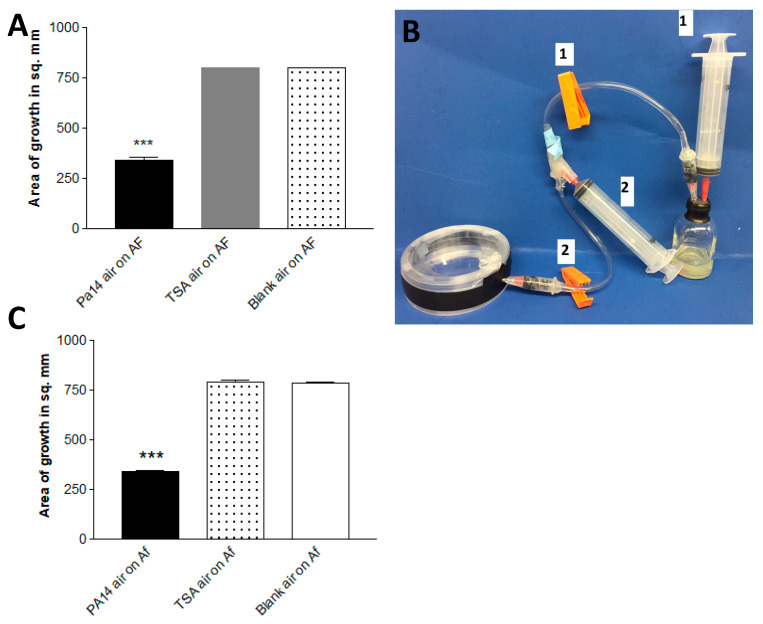
(**A**). Continuous production of Pa volatiles is not necessary for inhibition. Production from paired plates. After 48 h of incubation at 37 °C in the double plate method, air was aspirated via an 18-gauge needle from 2 paired plates of PA14 on TSA (Pa14 air), 2 paired uninoculated TSA plates (TSA air), or 2 empty plates (blank air), and the aspirated air was injected into 30 mL screw-cap glass vials with a rubber stopper containing 3 mL of TSA agar with 6 mm paper discs inoculated with 10 μL of a 5 × 10^4^ conidial suspension of 10AF. The vials were incubated at 37 °C for 72 h, and the area of fungal growth was measured. Three asterisks = *p* < 0.001 compared to other bars. (**B**). One-way air pump to aspirate air from the double plate, a method designed to capture the products of a smaller generating system (a single plate). When clamp 2 is closed and clamp 1 is open, air is removed for the system by syringe 1, which is then removed. Then, clamp 2 is opened, clamp 1 is closed, and the air from the double plate is aspirated by syringe 2. Then, clamp 2 is closed, clamp 1 is opened, and the air in syringe 2 is injected into the vial. (**C**). Production of sufficient inhibitory vapors from a single plate. Plates of TSA inoculated with PA14, uninoculated TSA, or an empty plate were paired in each case with an empty plate. This method was similar to that in Figure 3a, but instead of aspiration with a needle and syringe, we used the apparatus pictured in Figure 3b. Three asterisks = *p* < 0.001 compared to the other bars.

In this experiment, a variant of the double plate method was used. We co-incubated pairs of plates in each of these three combinations: Pa on TSA, TSA with TSA, or two empty plates (each of these three groups in quadruplicate). At 48 h and 37 °C, the air was aspirated from each set of combined plates by piercing the parafilm wrap with a needle connected to a 20 mL syringe, then each air sample was injected into rubber-stoppered sealed glass vials which contained an Af inoculum on paper discs on TSA (Figure 3a). After 72 h of incubation, the air extracted from the two combined Pa plates inhibited (*p* < 0.001) Af growth >50% compared to air extracted from either of the controls.

In a second double plate experiment, a single Pa-inoculated plate, a single uninoculated TSA plate, and a blank plate were each combined with a blank plate, and at 48 h the air content was pumped directly (Figure 3b) from each double plate into vials with Af, as in the first experiment. The results were the same (Figure 3c). These experiments demonstrate that the continuous production of Pa volatiles in the presence of Af is not necessary to inhibit Af (and that enough volatiles can be collected from a single plate to inhibit).

#### 3.4.4. Mechanism of Inhibition

**Figure 4:**
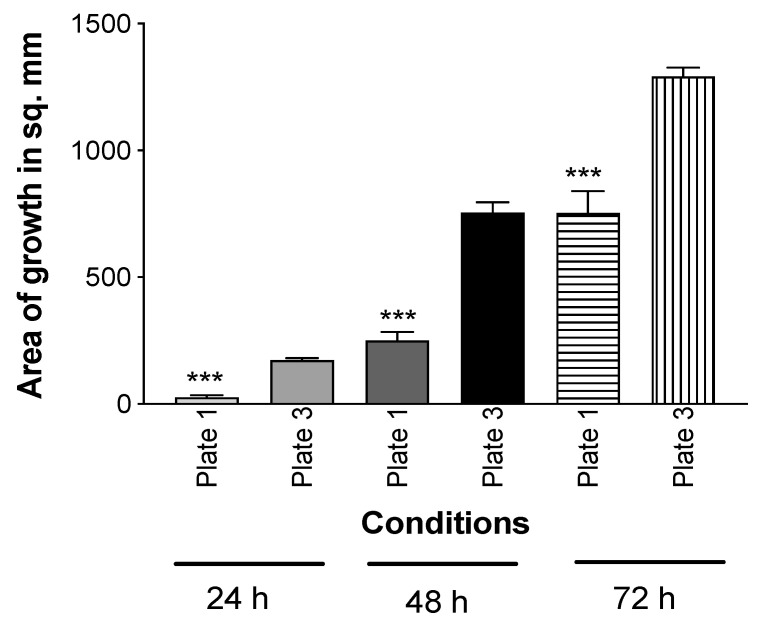
Mechanism of inhibition is by the absorption of inhibitory Pa molecules into agar. In the double plate method, TSA was inoculated with Pa10 (40 μL of a 10^9^/mL suspension) (plate #2) and paired with an uninoculated TSA plate (plate #1). An uninoculated TSA plate (plate #3) was paired with another uninoculated plate (plate #4). These pairs were incubated 72 h at 37 °C. Then, plates #2 and 4 were removed, and #1 and 3 were inoculated with 10 μL of a suspension of 5 × 10^4^ 10AF conidia, covered, incubated at 37 °C, and the areas of fungal growth calculated. Three asterisks = *p* < 0.001 compare the growth on plate 1, which was exposed to the aerosol products of Pa, to plate 3, which was exposed to only an uninoculated TSA plate. Times of incubation and measurement are indicated.

Using the double plate method, a TSA plate with Pa10 or an uninoculated TSA plate were incubated below uninoculated TSA plates for 72 h. At the conclusion of the incubation, the upper plates were removed and inoculated with Af (10AF). The results show that the uninoculated plate that had been incubated with Pa was now inhibitory to Af (Figure 4), indicating the volatiles had been absorbed into the agar and could still inhibit Af even though no Pa or Pa vapors were concurrently present.

This conclusion about mechanism was supported by another experiment where, in a double plate method, 5 × 10^7^ of 10AF conidia in PBS was exposed to a TSA plate inoculated with PA14 or to an uninoculated TSA plate (control). After 72 h, the conidia were harvested and serial dilutions of conidial suspensions were re-quantitated as CFU by inoculation onto PDA plates; also, undiluted conidia were inoculated onto RPMI agar and the growth areas were measured. The incubation with Pa volatiles neither affected the conidial number measured as CFU on PDA, nor the growth area resulting from the conidial inocula. This suggests that the volatiles do not directly affect Af.

### 3.5. Study of Pa Mutants

**Figure 5:**
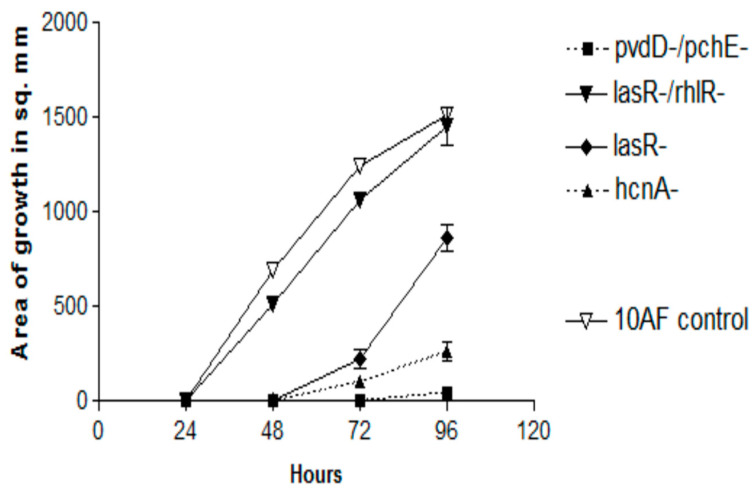
Effects of the production of volatiles by PA14 mutants on Af growth. Double plate method, as described in Figure 3a, with TSA plates. The magnitude of inhibition is demonstrated for the four mutants which were less active than the parent wild-type PA14. The complete inhibition by the remaining mutants (named in the text) and that of PA14 is not shown because they form lines of identity with the x axis for 96 h.

In previous studies [45,52,53], Pa mutants have been useful in dissecting the production of factors inhibitory to Af (in liquid media) (Supplementary Table S1). In the first double TSA plate experiment, all the Pa mutants grew well, and all the 26 mutants listed in the Supplementary Table S1 were as Af inhibitory as the PA14 parent, with the exception of these four mutants, which are listed from least inhibitory to more inhibitory: *lasR*-/*rhlR*- (no inhibition), *lasR*-, *hcnA*-, and *pvdD*-/*pchE*- (Figure 5). The *pvdD*-/*pchE-* mutant was insignificantly less inhibitory than the parent. The loss of activity in these four mutants and their order of loss of activity were confirmed in a second experiment, in which there was a small amount of inhibition seen with the *pvdD*-/*pchE*- mutant only at 96 h, and no significant differences were seen between it and the *hcnA-* mutant at any time. Thus, the two important mutations, *lasR*-/*rhlR-*, particularly, and *lasR*-, which resulted in the impaired production of the inhibitory volatiles, involved deletions resulting in the loss of several quorum sensing-related factors. A third experiment examined some Pa mutants from the PAO1 background. This *lasR*-/*rhlR-* mutant was again insignificantly inhibitory, whereas *pvdD*-, *pqsA-* (see Supplementary Table S1 for the notation of the defects of these three mutants)*,* and *coaB-* and the PAO728- mutants (58) of PAO1 were as inhibitory as the parent.

This discovery of a Pa mutant strain *(lasR*-/*rhlR-)* with unimpaired growth on TSA but lacking inhibitory volatiles was an important development, in that it created a control for the later analysis of the volatiles from inhibitory strains.

### 3.6. Study of Clinical Isolates

**Figure 6:**
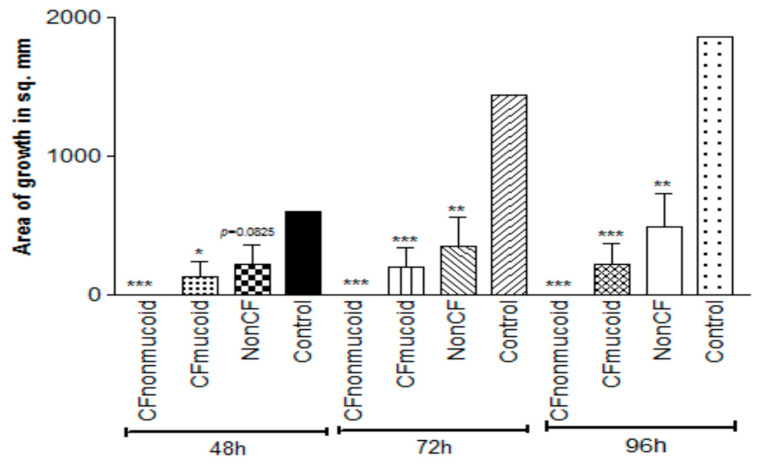
Studies of the volatiles of Pa clinical isolates. Double plate method, as described in Figure 3a, with TSA plates. Fifteen representative isolates, 5 in each group, were studied, and the aggregated results are presented. CF isolates were more inhibitory than the non-CF isolates, and the nonmucoid CF isolates were more inhibitory than the mucoid CF isolates at all the time points. Three, two, and one asterisk represent *p* < 0.001, *p* < 0.01, *p* < 0.05, respectively, compared to the control.

In a previous epidemiologic study, we found that Pa isolates varied significantly in their inhibition of Af in liquid, with non-mucoid CF isolates > mucoid CF isolates > non-CF isolates [40]. To compare the inhibition by volatiles, we selected five isolates from each group at random and compared them in five experiments on TSA against 10AF in the double plate method (Figure 6). At all three timepoints, inhibition of Af growth was complete for nonmucoid CF isolates, with the mucoid CF isolates intermediate and the non-CF isolates the least inhibitory. What is striking and perhaps unexpected about this finding is that the order of activity of these groups is the same as that seen in liquid media [40].

### 3.7. Is Inhibition by Pa Volatiles Restricted to A. fumigatus?

**Figure 7:**
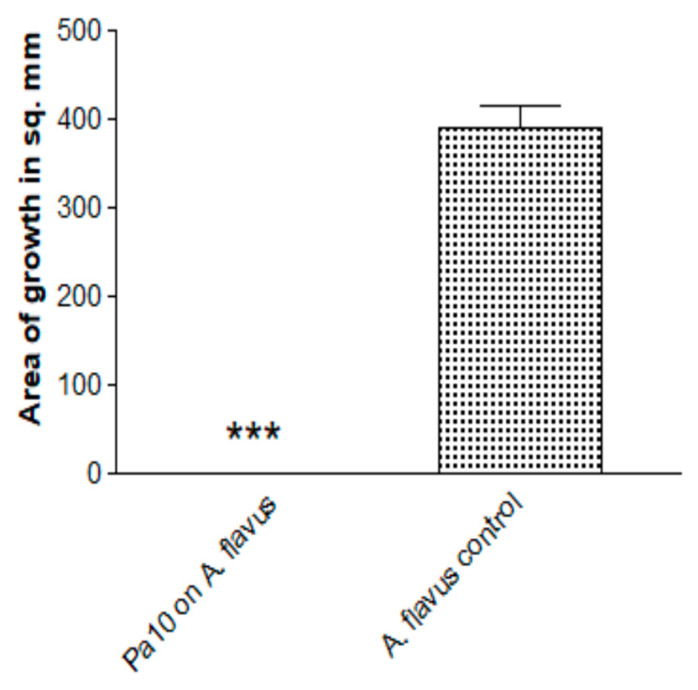
Inhibition by Pa of another Aspergillus species. *A. flavus*, isolate 10-103 (10 μL of 5 × 10^4^/mL conidial suspension), was studied with the double plate method (3 paper discs on 2 identical plates), TSA plates, Pa10 (40 μL of 10^9^/mL suspension), 48 h incubation, 37 °C. Three asterisks = *p* < 0.001.

We found that the inhibition of *Aspergillus* growth is not restricted to *A. fumigatus.* When *A. flavus* was similarly studied, the fungal growth was also inhibited (Figure 7).

### 3.8. Contrast with Reports That Pa Volatiles Stimulate Af

Because of reports [57,62] that Pa volatiles could stimulate Af, we performed tests with our system. The investigators [57] kindly donated the Af strain, CBS 144-89, used in their studies. In an initial double plate experiment, we studied Pa10, PA14, and PAO1 on TSA, comparing CBS 144-89 to 10AF, with readings at 24, 48, and 72 h. The CBS 144-89 grew identically (size) to the 10AF in the absence of the Pa. All three Pa inhibited both the Afs completely at all the time points. PAO1 is the Pa isolate that was previously reported [57] to stimulate.

**Figure 8:**
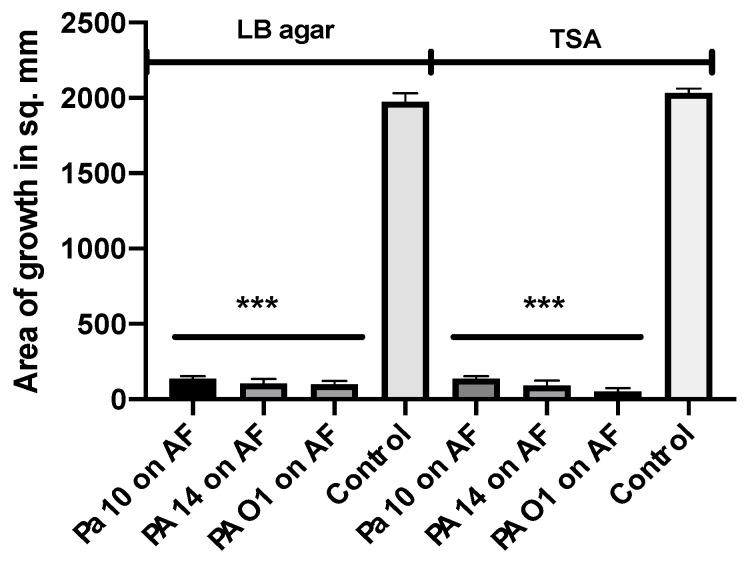
Study of a previously described method for studying the Pa volatile interaction with Af. The plate-in-plate method was used [57]. Three Pa strains (20 μL each from a 10^9^/mL suspension) were studied with 2 agars (LB agar, TSA), and the Af strain CBS 144-89 (5 μL of a 10^7^/mL suspension) was used, with incubation for 5 days at 37 °C. The corresponding agar type was used in the inner plate and the larger plate in each pairing. Three asterisks = *p* < 0.001.

We have described above that LB agar, one of the media used by these investigators [57,62], does not by itself produce volatiles stimulating Af growth. In another experiment, we compared the Af and Pa growth on both TSA and LB agar, the three Pa described in the above experiment, and CBS 144-89, but used the “plate-in-plate” method described by the investigators [57], including the micro-organism inoculum sizes they used. After five days of incubation, all three Pa inhibited the Af on both agars (Figure 8). This method, from the literature, is thus a fifth method of study that can be used for demonstrating inhibition.

### 3.9. Capturing of Volatiles in Liquid

#### 3.9.1. Effect of Trapping Materials

**Figure 9:**
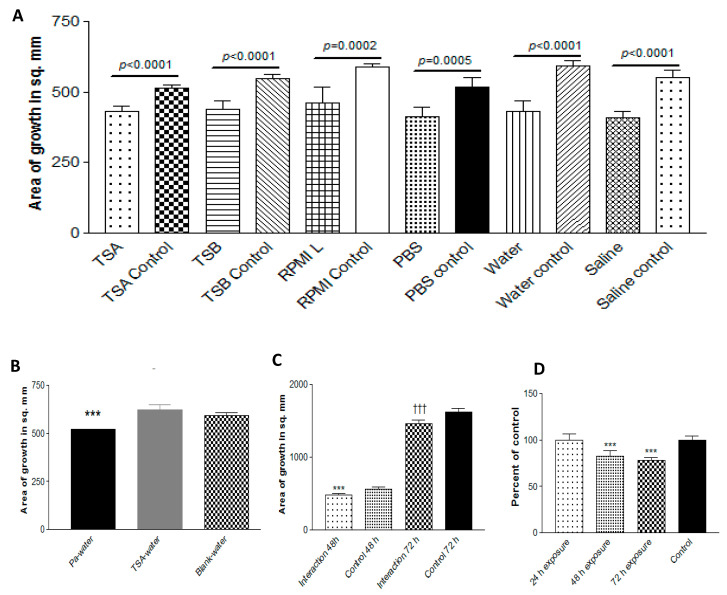
Capture of volatiles. (**A**). Trapping materials. Pa10 was grown on TSA for 72 h at 37 °C in the double plate method, with a second plate (trapping phase) as indicated. With a delay of <3 sec, the Pa plate was replaced with a plate of TSA with 10AF inoculum (exposure phase), incubated 48 or 72 h, and the growth was measured. The 48 h exposure data is shown. Unexposed plates (controls) are compared to exposed plates. (**B**). No inhibition of growth from an uninoculated plate. In a double plate experiment similar to that in Figure 9A, 40 μL from a Pa10 10^9^/mL suspension on TSA, or an uninoculated TSA plate, were paired with 15 mL of deionized water in the second plate, and incubated for 72 h at 37 °C. The 2 water plates were each then sealed in a second double plate setup with a TSA plate inoculated with 10 μL of 5 × 10^4^/mL conidial suspension of 10AF, incubated for 48 h at 37 °C, and the fungal growth measured. Three asterisks = *p* < 0.001. (**C**). Effect of time of exposure. Pa10 (40 μL from a 10^9^/mL suspension) inoculated on TSA plates, or uninoculated TSA plates (control), were incubated for 48 h at 37 °C in the double plate setup with 15 mL of water in the second plate (trapping phase). In the second part of the experiment, the double plate setups contained TSA plates inoculated with 10 μL of 5 × 10^4^/mL conidial suspension of 10AF, with the second plate containing the water that had been exposed to the Pa volatiles. This was incubated for 24 h at 37°C. Then, 24 and 48 h later, the water was replaced with water that had just trapped the Pa volatiles for 48 h. After 48 and 72 h of total co-incubation, the fungal growth was measured. “Interaction” refers to the cumulative time of 24 h of co-incubations of Af with the water that had trapped the Pa volatiles. Three asterisks = *p* < 0.001 compare the left 2 bars, and 3 daggers = *p* < 0.001 compare the right 2 bars. Refreshing the exposure of Af with fresh water that had trapped Pa volatiles maintains the inhibition over a 72 h period. (**D**). Optimal trapping duration. In the double plate method, a plate of deionized water was combined with a plate of Pa10 on TSA for 24, 48, or 72 h, and each water plate was then tested in exposure to 10AF on TSA.

To define the volatiles involved, we needed to ascertain how they might be captured. Using the double plate method, Pa10 on agar was grown with a second plate containing either TSA, TSB, PBS, RPMI, de-ionized water, or normal saline (trapping phase). Then, the Pa plate was quickly replaced with a plate with a 10AF inoculum (exposure phase) and the double plate was incubated (Figure 9a). All the trapping materials were able to inhibit 10AF in the absence of Pa (Figure 9a); the volatiles had not been lost in the brief delay, and the inhibitory volatiles could be re-released from the trapping materials. This experiment was repeated three times, with identical results. An uninfected TSA plate combined with water in the double plate did not result in any inhibitory properties in the water (Figure 9b), and in a repeat experiment, there was more pronounced stimulation of Af by water that had trapped volatiles from an uninoculated TSA plate, as if some nutrients for Af can volatilize from an uninoculated TSA plate.

#### 3.9.2. Effect of Time of Exposure of Af

The exposure period of 72 h with trapped volatiles had resulted in less Af inhibition than 48 h of exposure (waning effect, possibly owing to volatile dissipation or the deterioration of the volatile activity). To assess this, in another experiment, during the exposure phase, a fresh Petri plate of water that had just trapped volatiles for 72 h from Pa on TSA was put under the Af-TSA plate and replaced by another such fresh plate at 24 and at 48 h of exposure, and the effect was compared to water containing trapped volatiles that had been in the double plate for the whole 72 h exposure phase. With the replacements by the fresh captured volatiles, there was no waning effect when the Af growth was then assessed, indicating that the introduction of freshly trapped volatiles every 24 h during Af exposure could maintain the level of Af inhibition without waning (Figure 9c).

#### 3.9.3. Effect of Duration of Trapping

To assess the optimal duration of trapping, a plate with water was combined in the double plate method with a plate of Pa for 24, 48, or 72 h, and each water plate was then tested in exposure to Af. The optimal trapping duration was 72 h (Figure 9d). This was confirmed in a second experiment.

In these studies, we discovered that the trapping of volatiles led to a net alkalinization in the trapping materials, a possible clue to their trapped content. The pH of the deionized water was 5.5; the water in a plate combined with uninoculated TSA in the double plate method for 72 h had a pH of 5.5, whereas the water in a plate combined with Pa10 on TSA in the double plate method for 72 h had a pH of 7.5.

#### 3.9.4. Efficacious Trapping of Volatiles

**Figure 10:**
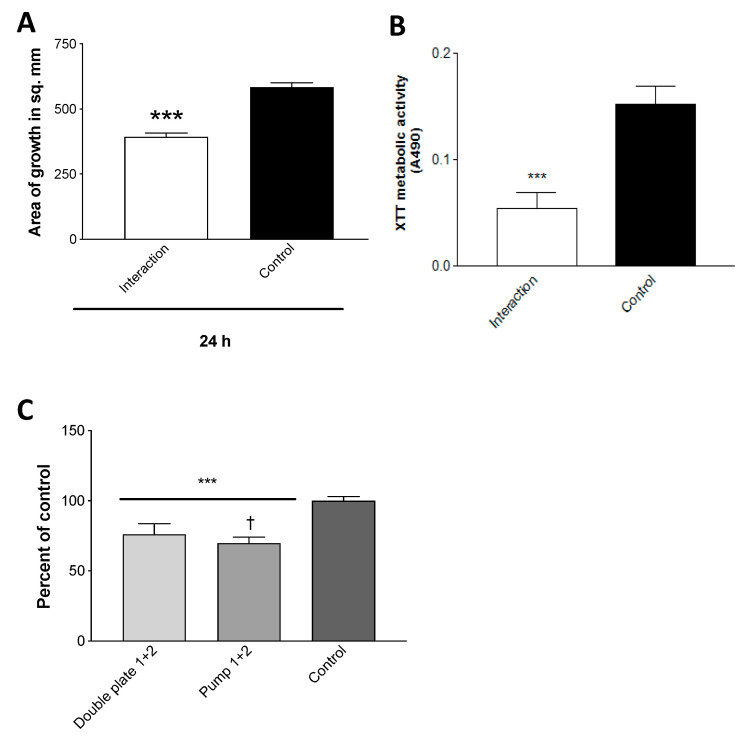
Efficacious trapping of volatiles. (**A**). Trapping. Air was pumped through tubing from an inoculated Pa10 (80 μL from a 10^9^/mL suspension), or from an uninoculated (control) 250 mL TSA plate, incubated for 72 h at 37 °C, bubbling through 25 mL of de-ionized water. The collected waters were then incubated in the double plate method with TSA plates inoculated with 10 μL of 5 × 10^7^/mL of conidial 10AF suspension for 24 h, and the growth was measured. “Interaction” refers to the water that had been exposed to Pa vapors, three asterisks = *p* < 0.001. (**B**). Trapped volatiles are inhibitory to Af metabolism as well as growth. The effects of the volatiles trapped in deionized water and control, described in the legend for Figure 10A, were studied for their effects on 10AF biofilm formation with the methodology detailed previously [40,43,44,45,46,52,53,54,58]. In this instance, parafilm was used to seal the 96-well plate, the plate lid was applied, and the plate was put in a plastic bag, with the aim of retaining, if necessary, the volatiles in the wells (in addition to those trapped in the water). “Interaction” refers to the water that had been exposed to Pa vapors, three asterisks = *p* < 0.001. (**C**). Comparison of de-ionized water trapping of volatiles by the double plate method and air pumping method. The water from 2 double plate experiments, trapping phase, was prepared from the incubation with the Pa10 on a TSA plate, as described in Figure 9A,B, and tested (“Double plate 1 + 2”). Two pumping methods were assayed for trapping of volatiles, one as shown in Figure 3b, the other as described in the text for the generation of Figure 10C (“Pump 1 + 2”). Controls were as previously described, the water was exposed to an uninoculated TSA plate. Growth of 10AF on a TSA plate was assayed (as detailed in the Figure 3a legend) after exposure in a double plate method to the trapped volatiles in water. The effects on growth from the water of the 2 double plate experiments were identical, and the results were pooled. The results from the water of the 2 pumping methods were identical, and their results were pooled. Comparisons are shown as a percent of the control. Three asterisks = *p* < 0.001 compare either of the 2 left bars to the control. One dagger signifies *p* < 0.05, comparing the 2 left bars. Pump methods of obtaining a gas phase containing volatiles from a Pa culture are modestly but significantly superior to aspiration via needle from double plates for obtaining the materials inhibitory to Af.

The double plate method would not directly allow the analysis of the volatile components. We have described above, in studies showing that the continuous production of volatiles is not necessary for inhibition, that the air between double plates can be aspirated or that the air could be pumped and inhibit Af in vials. This could also be a method to capture volatiles that are thus assuredly inhibitory for spectral analysis. To explore trapping volatiles in larger volumes for subsequent analysis and possibly more simply, we also assessed pumping air from a Pa-inoculated TSA plate through tubing and bubbling it through de-ionized water. The volatiles from the collected water were then inhibitory to the Af growth (Figure 10a); the assay was used to assess the inhibition in our studies, but also, for comparison, was studied for the inhibition of the Af biofilm metabolism (Figure 10b) (a metric for the inhibition of Af by Pa used more frequently than growth inhibition) [38,40,43,44,45,46,52,53,54,59] via the 96-well plate setting (described in Methods), and the inhibition was demonstrated. With respect to the growth inhibition by volatiles, the water resulted in a comparable Af inhibition to what can be seen with the contents of water trapped via the double plate method (Figure 10c).

### 3.10. Storing Trapped Volatiles for Mass Spectroscopy

Having studied the variables involved in trapping volatiles, it was necessary to ascertain the preservation of inhibitory activity, with a view toward storing and transporting the volatiles for mass spectroscopy. A series of five experiments was performed which examined the volatiles trapped in de-ionized water for 72 h and then stored either at room temperature; in the refrigerator (4 °C); or frozen (−20°C) for 24, 48, or 72 h. The materials stored in the refrigerator or frozen were brought to room temperature before testing. This study was an attempt to mimic the shipping conditions that might be necessary.

The TSA plates were inoculated with Pa10, or uninoculated (the control for comparison), incubated for 72 h in the double plate method with the waters, and the waters then tested for the volatile release of inhibitory activity against 10AF after storage under the several conditions just described, in a second double plate method.

In summary, no significant loss of activity of the trapped volatiles was noted under any of the three conditions, when compared with freshly generated volatiles trapped in water for 72 h. The volatiles from all the preparations inhibited the Af growth ~25% compared to the controls (plates with no water exposure). These results speak to the stability of the volatiles under the several temperature conditions described, as well as after freeze-thawing. However, when the water with trapped volatiles was instead heated in a sealed container at 58 °C for 30 min, there was a 13% deterioration (*p* < 0.001) in the inhibitory activity from this water, comparing the Af growth areas after exposure to a comparably prepared unheated preparation.

### 3.11. Mass Spectroscopy of Inhibitory Materials

Four studies were performed. In the first of these, three samples were prepared with the double plate method for 72 h at 37 °C. Deionized water was paired with either plates of Pa10 on TSA or TSA alone. As a third control, water alone was incubated as described. As another control, a fourth sample—distilled water used in the flushes of samples used for the mass spectroscopy machine—was studied.

In the second study, PA14 and the (non-inhibitory) *lasR-/rhlR-* mutant were studied as just described with the same sets of controls.

In these two studies, spectroscopy peaks appearing only in the test samples, and not in the controls or water exposed to the mutant, were considered to be potentially inhibitory volatiles of interest. It was considered that only water-soluble inhibitory volatiles would be captured and identified in these studies.

In the third study, a similar double plate method was used, but instead of the second plate of water, both plates of the pair were identical (to augment the collection of volatiles). These double plates were combinations of two plates: PA14 on TSA, the *lasR-/rhlR-* mutant on TSA, two uninoculated TSA plates, or two empty plates. At the end of the 48 h of incubation, the air that had collected in each pair was aspirated via a needle and one-way air pump into mass spectroscopy vials. An example of a spectroscopy tracing from this study is shown in the Supplementary Figure S1.

In the fourth study, the vials were coated with 5 mL of TSA and inoculated with either PA14 or the *lasR-/rhlR-* mutant (40 μL of a 10^9^/mL inoculum in saline), or a TSA-coated vial or an empty vial was given 40 μL of saline. All the vials were incubated as described above. With the live cells still present in the Pa vials, the air content of all the vials was sampled for mass spectroscopy.

In the latter two studies, the peaks present in the Pa vials but not in the three controls in each experiment were considered the inhibitory volatiles of interest, which now included the volatiles that were not water-soluble.

From the spectrographs resulting from these four studies, tables were prepared from the peaks identified: Table 1 shows the peaks excluded from interest because they had never been seen in the products of the inhibitory Pa isolates; Table 2 shows the peaks of less interest because they were seen in the controls as well as in the products of the inhibitory Pa isolates; and, finally, Table 3 shows the peaks of intense interest because they had been seen only in the products of the inhibitory Pa isolates and not in the controls. We then largely studied those molecules in Table 3 that we could obtain in pure form.

### 3.12. Studies of Molecules of Intense Interest as Inhibitory Volatiles

In the next series of 15 experiments, we studied those Table 3 molecules that could be obtained. DMSO was used as the universal solvent that was compatible with all of them. In the course of these studies, we compared the vial-in-jar method to the double plate method as the source of the vapors from the liquid molecule vessels, the volume of DMSO for the molecules to produce the maximal effect, and TSA agar vs. RPMI agar as the optimal milieu for the fungus being challenged. We found 1 mL of DMSO in a vial in the vial-in-jar method to be optimal, and TSA agar for the fungal plating to demonstrate maximal inhibition. With this large a volume of DMSO, we noted that DMSO itself could produce volatiles with a slight stimulatory effect on Af growth, so it is essential to have it as a comparator for the control when studying molecules with this scheme. The DMSO in the vials was noted to apparently be hygroscopic during the incubation, as the volume of the DMSO (with or without the test molecules added) in the vial slightly increased during the incubation period, presumably reflecting the capture of moisture from the air in the double plate.

**Figure 11:**
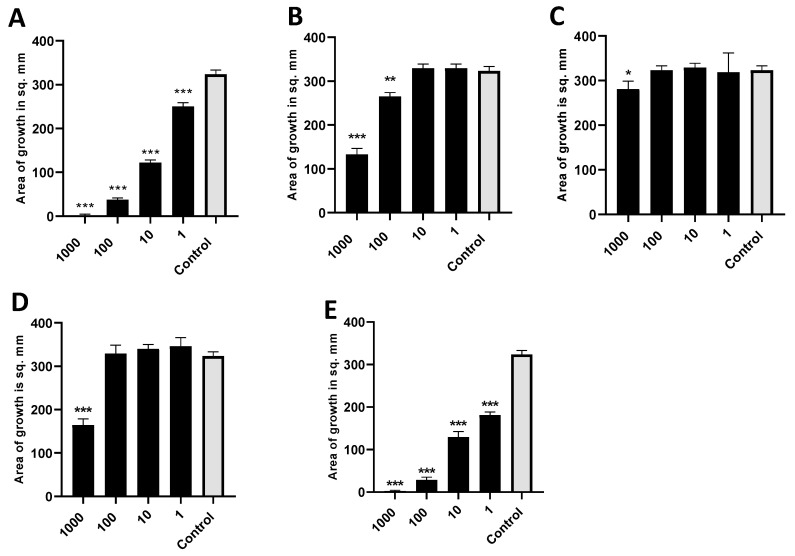
Activity of individual molecules identified in mass spectroscopy. (**A**). Dose titration of 2-heptanone on the growth area of 10AF. Vial in jar method was used, with incubation for 40 h at 37 °C. Control was DMSO, the same volume as DMSO with 2-heptanone (black bars). Numbers on the x axis are in mM. Three, two, and one asterisk represent *p* < 0.001, *p* < 0.01, and *p* < 0.05, respectively. Representative experiment of 6 replicate experiments. (**B**). Dose titration of 2-undecanone. Same methodology and display as 11a. Representative experiment of 2 replicate experiments. (**C**). Dose titration of 1-undecanol. Same methodology and display as 11a. Representative experiment of 2 replicate experiments. (**D**). Dose titration of 2-dodecanone. Same methodology and display as 11a. Representative experiment of 2 replicate experiments. (**E**). Dose titration of methyl thiobutyrate. Same methodology and display as 11a. Representative experiment of 3 replicate experiments.

We found 2-heptanone, 2-undecanone, and methylthiobutyrate from Table 3 each alone to be strongly active as inhibitors of *A. fumigatus*. 1-undecanol and 2-dodecanone produced only a slight inhibitory effect. These results are shown in Figure 11. In similar studies, 2-undecanol or 2-tridecanone had no inhibitory effect. We also studied the important Pa quorum-sensing molecule, N-(3-oxododecanoyl)-L-homoserine lactone (3-OC_12_-HSL), and the Pa siderophore, pyoverdin, in the same way, and they also had no inhibitory effect as volatiles on *A. fumigatus*.

**Figure 12:**
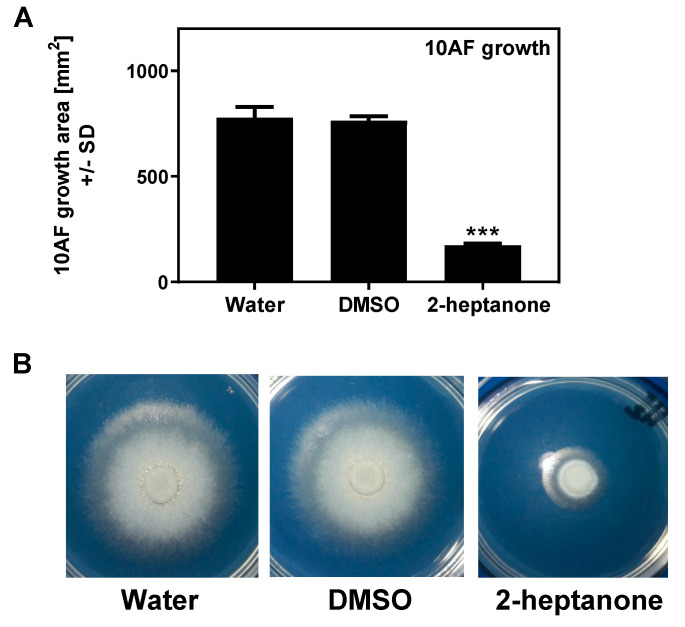
Preconditioning of agar plates with 2-heptanone interferes with 10AF growth. TSA plates were exposed to either sterile water, DMSO, or 2-heptanone (5.7 M), on 1 cm paper disks in lids for 90 min at room temperature, followed by the removal of the agents. One filter disk was placed on each pre-conditioned agar, inoculated with 10 µL of *A. fumigatus* suspension (5 × 10^4^ conidia/mL), and the sealed plates were incubated at 37 °C for 48 h. (**A**) 10AF growth areas after 48 h of incubation; 3 plates were used for each condition. Three asterisks represent *p* < 0.001, compared to the water control. (**B**) One of each of the plates used in A was used as an example to visualize the 10AF growth under each condition.

In a previous study (Figure 4), we found evidence that a TSA plate that had been exposed to the vapors of Pa was inhibitory to Af, even with Pa nor vapors no longer present, indicating that the mechanism of inhibition was absorption into the agar of inhibitory molecules. We repeated this study using 2-heptanone, one of the most inhibitory molecules, which gave the same result: a TSA plate that had been incubated with the volatile was inhibitory to Af in the absence of the volatile (Figure 12).

### 3.13. Combinations of Inhibitory Molecules

**Figure 13:**
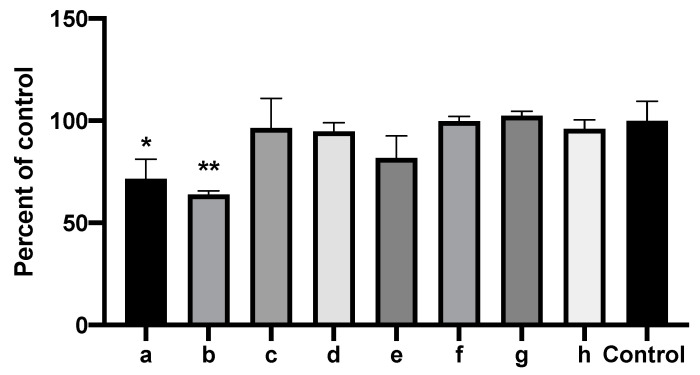
Combinations of inhibitory molecules identified in mass spectroscopy are not synergistic. Vial in jar method, as per Methods. One milliliter of 10 mM of each of the chemicals to be tested in DMSO or 1 mL DMSO alone (“Control”) were placed in the vials. Fungal growth area with the test chemicals was compared to the control (percentages). Two and one asterisks represent *p* < 0.01 and *p* < 0.05, respectively, compared to the control. a through h are, respectively, 2-heptanone, methyl thiobutyrate, 2-nonanone, 2-heptanone + methyl thiobutyrate, 2-heptanone + methyl thiobutyrate + 2-nonanone, combination of all the chemicals mentioned except 2-heptanone + methyl thiobutyrate, combination of all the chemicals except 2-heptanone + methyl thiobutyrate + 2-nonanone, and combination of all the chemicals mentioned combined.

We anticipated that the combinations of inhibitory molecules could be synergistic (Figure 13). However, to our surprise, the combination of 2-tridecanone, 2-undecanol, 2-undecanone, 1-undecanol, and 2-butanone (the latter a molecule found to be inhibitory alone, but that had also been seen in control vapors) had no inhibitory activity, despite the activity individually shown for all but the first two in this mixture (all the molecules, in these studies, were tested together in concentrations, 10 mM, of each that had been inhibitory alone in prior studies). Similarly, the above combination plus 2-nonanone (another molecule that was inhibitory when tested alone, but that was also seen in control vapors) was also non-inhibitory. We tested a combination of two inhibitory molecules, 2-heptanone and methylthiobutyrate, and found that the combination was significantly inhibitory, but less so compared to the molecules tested individually. Finally, we tested in this manner 2-heptanone, methylthiobutyrate, and 2-nonanone, three molecules that were individually inhibitory, and again found the combination to be significantly inhibitory, but less so compared to the molecules tested individually (Figure 12 and Figure 13). We conclude from this group of studies that the molecular interactions are complex, that molecules may interfere with each other in the liquid (interfering with the release of volatiles), that molecules may interfere with each other’s diffusion to the target after capture in the nutrient substrate (agar), and/or that they may interfere with each other in the mechanism of inhibition at the fungal target.

## 4. Discussion

Pa and Af are the most important bacterium and fungus in the microbiota in many clinical settings, particularly in the lungs of immunocompromised persons and those with CF. Advanced technology has been used to detect the volatiles of Pa and other microbes [57,63,64,65,66,67,68,69,70,71,72,73,74,75], and such technology is currently under study as methods for the noninvasive detection of these pathogens [66,72,73,74,75,76]. Nearly 400 volatiles have been described from Pa, of which ketones are the most highly represented [71]. Here, we add simple methods to the previously published methods [57] for studying intermicrobial volatile interactions, which can be adopted by other laboratories to study the interface, potentially clinically important, between micro-organisms in the human microbiome. We have also provided data on the different trapping materials, methodologies, and temporal metrics that could be helpful in further studying volatiles in the intermicrobial interaction. Whereas it is possible there could be differences in which volatiles are produced by Pa over time in culture, a lack of qualitative changes has been noted after the first 24 h in culture [64]. The testing and development here, for the availability of useful and practical methodologies, is particularly important because of the next series of questions that need to be addressed by researchers, discussed below, and because of the differences found in the volatile intermicrobial interactions to date [57,62] that should be resolved.

It was notable that the volatiles of Pa isolates from CF patients, particularly non-mucoid Pa isolates, were more inhibitory than others (as was reported for the inhibition of Af in liquid medium) [40]. The latter studies indicated that pyoverdin was responsible for the inhibition differences described [40,45,52,53], at least in iron-limited settings. These properties of CF isolate products in liquid and vapor have clinical implications for intermicrobial interactions in CF airways.

One concern in studies in closed systems, such as those previously described [57] or our double plate method, is that the two actors could be enclosed in a hypoxic environment, which certainly would affect their interactions [43]. However, comparing our experiments- examining the Af growth in conditions where an empty plate, an uninoculated plate of medium, or no second plate were studied; studies where Pa volatiles were pumped into receptacles with Af growing or that were collected and later exposed to Af; similar studies where air between double plates was aspirated and then tested for inhibition; studies where Af plates were not introduced until later times in incubation; and studies of noninhibitory Pa mutants which grew robustly in co-cultures- all these studies, which indicated that the Af growth was unimpaired compared to when inhibitory Pas were present, show that it was the Pa volatiles and not hypoxia that contributed to the Af inhibition. Nor were the characteristics of Af growth in hypoxic environments [43] observed in those of our systems that were closed (but that of course contained some ambient air at the outset of incubation). That the mechanism of inhibition appears to be incorporation of volatiles into the agar, and not action involving the contact of volatiles with developing Af structures, would also be consistent with concluding that hypoxia is not a factor in our observations. Another important finding from the experiment showing that the volatiles are captured in agar and are inhibitory even when Af is introduced into the setup later is that it indicates that the volatiles are constitutively produced by Pa, and do not require the presence of Af as the stimulant.

Microbial volatiles are typically small lipophilic organic molecules [77]. The mechanism of inhibitory action of such volatiles has been ascribed to interactions with the hydrophobic segments of proteins [78]. Regarding our observations with the quorum-sensing mutants, that the *lasR*-/*rhlR*- mutant was noninhibitory, the *lasR-* mutant was only somewhat inhibitory, and the *rhlR-* mutant was fully inhibitory, opens the possibility of now defining which downstream pathways in Pa are responsible for these differences.

Af also produces volatiles [68,69,70] (and GC-MS has been recommended to sort them out, as we have done in our studies with Pa volatiles) [69,70]; we have not noted the effects on Pa growth in co-cultures compared to the controls just described, but we have not yet systematically studied that. As we reported here, there was some Af stimulation by vapors from an uninoculated agar plate.

There are several shortcomings of this study. We have not pinpointed all the Pa molecules that could be involved in Af inhibition, as some candidates that were identified by chromatography studies could not be obtained for separate study. A larger issue is how inhibitory molecules interact with each other; it was highlighted by our observations that commonly the sum of the parts was less than expected from the results of the individual components, and that noninhibitory molecules could affect the results of inhibitory molecules. It is also possible that the net Af-inhibitory effect we have described is a result of the interactions of volatiles produced by Af and Pa, an interaction that may be of a synergistic or antagonistic nature; or that one actor may modify the molecules produced by the other [49]; or that some volatiles may stimulate biofeedback loops in one or both participants, reacting to the volatiles they sense and modifying their behavior [79]. In addition, more sample preparations, such as liquid-liquid extraction prior to the GC-MS analysis, or the use of liquid chromatography-MS, might have identified more molecules, especially those that are less volatile, more polar, or have a higher molecular weight. Furthermore, our selection of candidates relied on excluding the molecules seen in controls, including the volatiles from noninhibitory Pa. We cannot conclude either that our candidates (Table 3) were not also present in controls but below detection, since the areas under the peaks detected by the chromatographic method are only a semiquantitative indicator. We cannot assess the relative contributions of the candidate molecules detected for the same reason, nor whether the molecules detected by us from the vapors of an uninoculated plate, or detected from a plate inoculated with a noninhibitory Pa, might also be present in volatiles from an inhibitory Pa and contribute to inhibition via some synergistic interaction. Our mixing of pure molecules in equal concentrations of each in testing the inhibitory power of the volatiles from a mixture also may not reflect their relative concentrations present in the vapors from a Pa colony growing on an agar plate. Finally, in a complex milieu, such as a CF lung, the molecules produced by microbes other than Pa or Af may influence the volatile production by, or volatile effects on, Pa and/or Af. It would also be of interest in future investigations to detail the morphological effects of volatiles on Af, such as mycelial branching, septation, cell wall thickness, nuclei number, etc.

Many of the volatiles of interest to us as potential Pa inhibitors (Table 3) have been noted previously among Pa volatiles, including 2-undecanone [57,63,64,65,66,67,71,76], 2-heptanone [64,65,66,67,71,76], and methyl thiocyanate [67]. 2-heptanone has been noted to be an inhibitor of micro-organisms (bacteria) [78]. An important confounder in volatile studies, as also noted by others [63], is that uninoculated culture medium produces volatiles that could be confused with those of the micro-organisms of interest; in those of our four chromatographic studies described which required such controls, we have noted many of these, and whereas the media themselves, when exposed to Af, were not inhibitory, those volatiles were excluded from our group of candidates as Pa inhibitors. A difference between our studies and previously published studies [57,62] that have reported that Pa volatiles stimulate Af is that we have never, in our multiple studies reported here, observed that phenomenon. The stimulation was attributed to sulfur-containing Pa volatiles. Dimethyl sulfide was implicated as a possible stimulant; we noted dimethyl sulfide as a Pa volatile, but it was also detected in a noninhibitory Pa mutant and thus was of less interest to us as a possible inhibitory volatile (Table 2). These other investigators reported four different media for Pa growth [57,62], used different media for Af growth, studied only one (or possibly two) different Af strains, and relied on a standard laboratory Pa strain [57,62]. One of the media used for Af, where the sulfur-dependent Pa stimulation of Af was reported, was sulfur-poor [57,62]. The formulae for some of the media they used was not stated or cited or was not readily available to us, but when we attempted to mimic the setup they used with one of the media they reported and with the Pa and Af strains they studied, we still observed only Af inhibition. These investigators also tested 2-undecanone and found that it was not inhibitory to Af [57], which was again in contrast to what we found. All these differences in findings are incompletely resolved, and require further study. An important observation in other prior studies is that there can be considerable variation in the production of different volatiles among various Pa strains [64,67,71,72]; culture conditions also affect the spectrum of Pa volatiles produced [61,67,72,76]. It is also possible that vapors from some of the Af media they used interacted with the Pa volatiles, which may explain the differences [57,62] from our results. They also noted that the stimulants appeared to act directly on the fungal structures and not by incorporation into the medium [62], which is the opposite acquisition phenomenon from what we report. We emphasize that our many studies reported in this paper have included observations with many different media, with several different Pa and Af strains, and with several methods of acquiring all the possibly relevant Pa volatiles.

Another important potential application of our findings is that specific volatiles, from micro-organisms such as *Pseudomonas*, may prove useful against fungi that are plant pathogens [80].

## Figures and Tables

**Table 1:** Molecules excluded because they were never seen from inhibitory Pa strains.

dimethyl trisulfide
3-methyl butanol
benzaldehyde
4 hydroxy-4-methyl-2-pentanone
pentanal
2-acetylthiazole
unknown ions at positions 41 and 57
unknown C10 H20 molecule

**Table 2:** Molecules excluded from intense study because they were seen in the volatiles of controls, or those from noninhibitory Pa strains.

acetone
2-butanone
isoamyl alcohol
2-nonanone
dimethyl sulfide
acetophenone
dimethyl disulfide
2,5-dimethylpyrazine
methyl methacrylate
toluene
ethyl benzene
xylene isomer
methoxy-phenyl-oxime
2-ethyl-1-hexanol
butylated hydroxytoluene
2(-1, 1, 3, 3-tetramethylbutyl)-phenol
methanethiol
isopropyl alcohol
thiopavalic acid
2-pentyl furan
1-undecene
2-amino acetophenone
unknown ions at positions 325 and 341
unknown ions at positions 41 and 6

**Table 3:** Molecules only ever seen from inhibitory Pa strains and not in controls.

methyl thiocyanate *
methyl thiobutyrate *
methyl thio isovalerate *
2-undecanone *
heptane
mercaptoacetone
5-methyl ester butane thioic acid
2-heptanone
decanone
2-undecanol
2-tridecanone
1-undecanol
2-dodecanone

* also determined to be water soluble (appeared in experiments described where de-ionized water was used as the trapping material).

## Supplementary Materials

The following are available online at https://www.mdpi.com/2309-608X/6/3/118/s1: Figure S1: Representative GC-MS tracing; Table S1: PA14 mutants.
