# Supplementary material for: Aspergillus Is Inhibited by Pseudomonas aeruginosa Volatiles
Source: J Fungi (Basel). 2020 Jul 25;6(3):118. doi: 10.3390/jof6030118 (PMC7557479; doi:10.3390/jof6030118)
Supplement: Supplementary file 1 [file jof-06-00118-s001.zip › Supplementary figure.pdf]

Supplementary figure

Representative GC-MS tracing

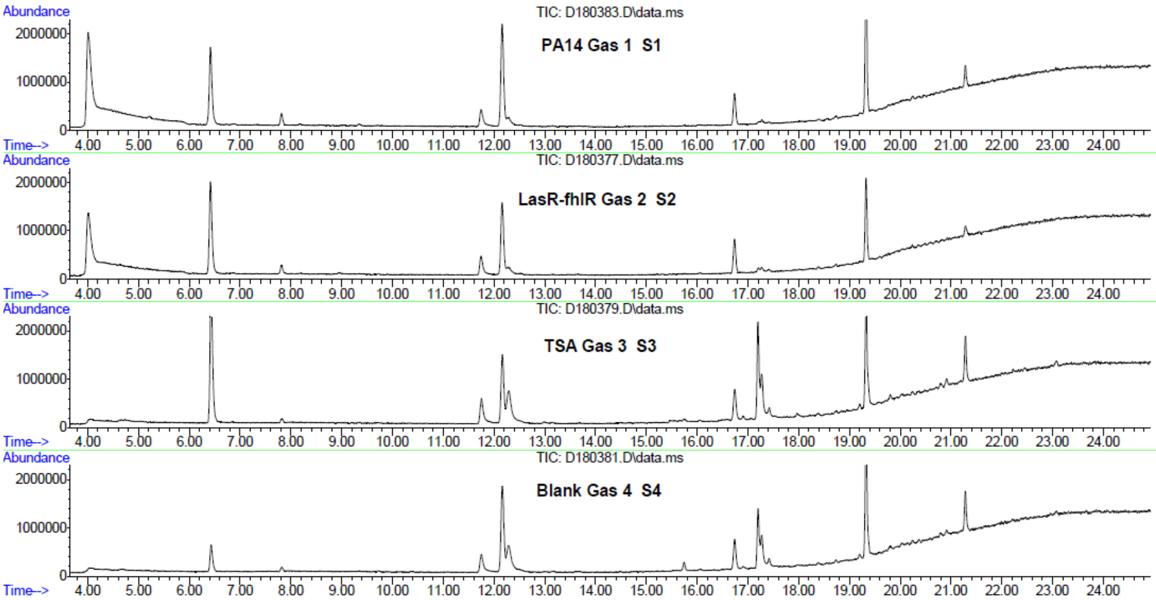

Chromatograph from part of the "third study" discussed in Results. The starting materials were the air ("Gas") phase from double plate apparatuses. From top to bottom, the tracings are from PA14 inocula, *lasR-/rhlR-* inocula, uninoculated TSA plate, and 2 empty plates, respectively. The peaks shown, and those from the 3 other studies not shown, are identified and arranged in Tables 1-3.
