# Supplementary material for: Aspergillus Is Inhibited by Pseudomonas aeruginosa Volatiles
Source: J Fungi (Basel). 2020 Jul 25;6(3):118. doi: 10.3390/jof6030118 (PMC7557479; doi:10.3390/jof6030118)
Supplement: Supplementary file 1 [file jof-06-00118-s001.zip › Supplementary Table PA14 mutants.docx]

**Suppl. Table: PA14 mutants** (previously published: Sass, G. et al., J. Bacteriol. 200(1):e00345-17, 2018.)

| **Mutant number** | **Mutant name** | **Mutation result** | **Ref.** |
| --- | --- | --- | --- |
| **1** | *pvdD-pchE-* | Pyoverdine-pyochelin double siderophore mutant | (^[[1]](#endnote-1)^) |
| **2** | *pqsE-* | Gene mediates the regulatory activity of the MvfR system (see *mvfR-*); HAQs similar to wild type but defective in pyocyanin, HCN | (^[[2]](#endnote-2)^) |
| **3** | *mvfR-* | Regulates the transcription of *pqsABCDE* operon; under-production of various QS-regulated factors related to loss of HAQs, phenazines, proteases, HCN, lectins and others (^[[3]](#endnote-3)^) | (^[[4]](#endnote-4)^) |
| **4** | *pqsA-* | First step in HAQ synthesis; gene for anthranilate-CoA ligase lost; loss of extracellular quinolones, HAQ biosynthesis, including HHQ, PQS, DHQ; also decreased activity of MvfR (^[[5]](#endnote-5)^) | (1) |
| **5** | *pqsH-* | Product converts HHQ into PQS; loss of 2-heptyl-3-hydroxy-4(1H)-quinolone synthase; loss of PQS biosynthesis, thus decreased activity of MvfR; MvfR not as negatively affected than for pqsA-, since pqsH- still produces HHQ and other HAQs that can act as ligands of MvfR | (^[[6]](#endnote-6)^) |
| **6** | *lasR-rhlR-* | Double mutant defective for most QS-regulated metabolites, including phenazines, rhamnolipids, AHL, HAQs, proteases, HCN, chitinase, elastase and others | (^[[7]](#endnote-7)^) |
| **7** | *lasR-* | Lacks several QS-regulated factors, including proteases, oxo-C12-HSL; delayed activation of *pqsABCDE* and of RhlR QS pathway | (1) |
| **8** | *rsmA-* | Global post-transcriptional regulator mutant; various effects including less rhamnolipids, more phenazines and HCN **(**^[[8]](#endnote-8)^) | (^[[9]](#endnote-9)^) |
| **9** | *pqsA-pqsH- NOT polar* | Same predicted outcome as pqsA-. In both, the *pqsA-* mutation is nonpolar on downstream genes in the operon, thus not preventing their transcription. | (5) |
| **10** | *pvdD-* | Loss of pyoverdine (siderophore) | (8) |
| **11** | *rhlR-* | Lacks several QS-regulated factors. Loss of rhamnolipids, phenazines, HCN, lectins, C4-HSL | (2) |
| **12** | *HSI-1/2-* | Double deletion mutant defective in 2 of 3 type VI secretion systems | (^[[10]](#endnote-10)^) |
| **13** | *pvcA-* | Loss of paerucumarin and pseudoverdin | (8) |
| **14** | *rhlA-* | Loss of rhamnolipids (viii) | (^[[11]](#endnote-11)^) |
| **15** | *phzC1-*  *phzC2-* | Double phenazine mutant (completely abrogated), no pyocyanin | (1) |
| **16** | *pchE-* | Loss of pyochelin (siderophore) | (8) |
| **17** | *exoU-* | Loss of exotoxin U, via type III secretion | (8) |
| **18** | *rsmY-,rsmZ-* | Loss of genes for coding small regulatory RNAs, antagonistic to RsmA;, decreased production of C4-HSL, phenazines, chitinase (vii) | (1) |
| **19** | *HSI-2/3-* | Double deletion mutant defective in 2 of 3 type VI secretion systems | (9) |
| **20** | *HSI-1/3-* | Double deletion mutant defective in 2 of 3 type VI secretion systems | (9) |
| **21** | *pqsA-pqsH- polar* | pqsA::TnPhoA, pqsH::Gm, the mutation in pqsA is polar (thus theoretically preventing transcription of downstream genes in the operon), Kan and Gm resistant | (1) |
| **22** | *chiC-* | Chitinase C mutant | (8) |
| **23** | *lecA-* | Lectin A mutant | (8) |
| **24** | *hcnA-* | Loss of hydrogen cyanide | (8) |
| **25** | *lasI-* | Impaired phenazine pathway, no oxo-C12-HSL release |  |
| **26** | *pscC-* | Defective in all 3 secretion systems |  |

Abbreviations: QS: quorum-sensing; HAQ: 4-hydroxy-2-alkylquinolones; HCN: hydrogen cyanide; AHL: acylhomoserine lactones; HSL: homo serine lactone; HHQ: 4-hydroxy-2-heptylquinoline; PQS: 3,4-dihydroxy-2-heptylquinoline (^[[12]](#endnote-12)^); HQNO:
4-hydroxy-2-heptylquinoline *N*-oxide. Kan: kanamycin; Gm: gentamicin.

1. Sass G, Nazik H, Penner J, Shah H, Ansari SR, Clemons KV, Groleau MC, Dietl AM, Visca P, Haas H, Déziel E, Stevens DA. 2017. [Studies of *Pseudomonas aeruginosa* mutants indicate pyoverdine as the central factor in inhibition of *Aspergillus fumigatus* biofilm.](https://www.ncbi.nlm.nih.gov/pubmed/29038255) J Bacteriol pii: JB.00345-17. doi: 10.1128/JB.00345-17. [↑](#endnote-ref-1)
2. Déziel E, Lépine F, Milot S, He J, Mindrinos MN, Tompkins RG, Rahme LG. 2004. [Analysis of Pseudomonas aeruginosa 4-hydroxy-2-alkylquinolines (HAQs) reveals a role for 4-hydroxy-2-heptylquinoline in cell-to-cell communication.](https://www.ncbi.nlm.nih.gov/pubmed/14739337) Proc Natl Acad Sci U S A 101:1339-1344. [↑](#endnote-ref-2)
3. Déziel E, Gopalan S, Tampakaki AP, [Lépine F](https://www.ncbi.nlm.nih.gov/pubmed/?term=L%C3%A9pine%20F%5BAuthor%5D&cauthor=true&cauthor_uid=15686549), [Padfield KE](https://www.ncbi.nlm.nih.gov/pubmed/?term=Padfield%20KE%5BAuthor%5D&cauthor=true&cauthor_uid=15686549), [Saucier M](https://www.ncbi.nlm.nih.gov/pubmed/?term=Saucier%20M%5BAuthor%5D&cauthor=true&cauthor_uid=15686549), [Xiao G](https://www.ncbi.nlm.nih.gov/pubmed/?term=Xiao%20G%5BAuthor%5D&cauthor=true&cauthor_uid=15686549), [Rahme LG](https://www.ncbi.nlm.nih.gov/pubmed/?term=Rahme%20LG%5BAuthor%5D&cauthor=true&cauthor_uid=15686549). 2005. [The contribution of MvfR to Pseudomonas aeruginosa pathogenesis and quorum sensing circuitry regulation: multiple quorum sensing-regulated genes are modulated without affecting lasRI, rhlRI or the production of N-acyl-L-homoserine lactones.](https://www.ncbi.nlm.nih.gov/pubmed/15686549) Mol Microbiol 55:998-1014. [↑](#endnote-ref-3)
4. Cao H, Krishnan G, Goumnerov B, Tsongalis J, Tompkins R, Rahme LG. 2001. [A quorum sensing-associated virulence gene of Pseudomonas aeruginosa encodes a LysR-like transcription regulator with a unique self-regulatory mechanism.](https://www.ncbi.nlm.nih.gov/pubmed/11724939) Proc Natl Acad Sci U S A 98:14613-14618. [↑](#endnote-ref-4)
5. [Wade DS](https://www.ncbi.nlm.nih.gov/pubmed/?term=Wade%20DS%5BAuthor%5D&cauthor=true&cauthor_uid=15968046), [Calfee MW](https://www.ncbi.nlm.nih.gov/pubmed/?term=Calfee%20MW%5BAuthor%5D&cauthor=true&cauthor_uid=15968046), [Rocha ER](https://www.ncbi.nlm.nih.gov/pubmed/?term=Rocha%20ER%5BAuthor%5D&cauthor=true&cauthor_uid=15968046),  Ling EA, [Engstrom E](https://www.ncbi.nlm.nih.gov/pubmed/?term=Engstrom%20E%5BAuthor%5D&cauthor=true&cauthor_uid=15968046), [Coleman JP](https://www.ncbi.nlm.nih.gov/pubmed/?term=Coleman%20JP%5BAuthor%5D&cauthor=true&cauthor_uid=15968046), [Pesci EC](https://www.ncbi.nlm.nih.gov/pubmed/?term=Pesci%20EC%5BAuthor%5D&cauthor=true&cauthor_uid=15968046). 2005. Regulation of Pseudomonas quinolone signal synthesis in Pseudomonas aeruginosa. [J Bacteriol](https://www.ncbi.nlm.nih.gov/pubmed/?term=Wade+DS+2005) 187:4372-4380. [↑](#endnote-ref-5)
6. Xiao G, Déziel E, He J, Lépine F, Lesic B, Castonguay MH, Milot S, Tampakaki AP, Stachel SE, Rahme LG. 2006. [MvfR, a key Pseudomonas aeruginosa pathogenicity LTTR-class regulatory protein, has dual ligands.](https://www.ncbi.nlm.nih.gov/pubmed/17083468) Mol Microbiol 62:1689-1699. [↑](#endnote-ref-6)
7. Dekimpe V, Déziel E. 2009. [Revisiting the quorum-sensing hierarchy in Pseudomonas aeruginosa: the transcriptional regulator RhlR regulates LasR-specific factors.](https://www.ncbi.nlm.nih.gov/pubmed/19246742) Microbiology 155:712-723. [↑](#endnote-ref-7)
8. Petrova OE, Sauer K. 2010. [The novel two-component regulatory system BfiSR regulates biofilm development by controlling the small RNA rsmZ through CafA.](https://www.ncbi.nlm.nih.gov/pubmed/20656909) J Bacteriol 192:5275-5288. [↑](#endnote-ref-8)
9. Liberati NT, Urbach JM, Miyata S, Lee DG, Drenkard E, Wu G, Villanueva J, Wei T, Ausubel FM. 2006. [An ordered, nonredundant library of Pseudomonas aeruginosa strain PA14 transposon insertion mutants.](https://www.ncbi.nlm.nih.gov/pubmed/16477005) Proc Natl Acad Sci U S A 103:2833-2838. [↑](#endnote-ref-9)
10. [Lesic B](https://www.ncbi.nlm.nih.gov/pubmed/?term=Lesic%20B%5BAuthor%5D&cauthor=true&cauthor_uid=19497948), [Starkey M](https://www.ncbi.nlm.nih.gov/pubmed/?term=Starkey%20M%5BAuthor%5D&cauthor=true&cauthor_uid=19497948), [He J](https://www.ncbi.nlm.nih.gov/pubmed/?term=He%20J%5BAuthor%5D&cauthor=true&cauthor_uid=19497948), [Hazan R](https://www.ncbi.nlm.nih.gov/pubmed/?term=Hazan%20R%5BAuthor%5D&cauthor=true&cauthor_uid=19497948), [Rahme LG](https://www.ncbi.nlm.nih.gov/pubmed/?term=Rahme%20LG%5BAuthor%5D&cauthor=true&cauthor_uid=19497948). 2009. Quorum sensing differentially regulates Pseudomonas aeruginosa type VI secretion locus I and homologous loci II and III, which are required for pathogenesis. [Microbiology](https://www.ncbi.nlm.nih.gov/pubmed/?term=Lesic+Starkey+He+Hazan+Rahme) 155:2845-2855. [↑](#endnote-ref-10)
11. Ochsner UA, Fiechter A, Reiser J. 1994. [Isolation, characterization, and expression in Escherichia coli of the Pseudomonas aeruginosa rhlAB genes encoding a rhamnosyltransferase involved in rhamnolipid biosurfactant synthesis.](https://www.ncbi.nlm.nih.gov/pubmed/8051059) J Biol Chem 269:19787-19795. [↑](#endnote-ref-11)
12. Lépine F, Déziel E, Milot S, Rahme LG. 2003. [A stable isotope dilution assay for the quantification of the Pseudomonas quinolone signal in Pseudomonas aeruginosa cultures.](https://www.ncbi.nlm.nih.gov/pubmed/12829259) Biochim Biophys Acta 1622:36-41. [↑](#endnote-ref-12)
